# Mapping scrub vegetation cover from photogrammetric point clouds: A useful tool in reserve management

**DOI:** 10.1002/ece3.7527

**Published:** 2021-05-01

**Authors:** Jim Vafidis, Isaac Lucksted, Moyrah Gall, Pete Maxfield, Kathy Meakin, Mark Steer

**Affiliations:** ^1^ University of the West of England Bristol UK; ^2^ Gloucestershire Wildlife Trust Gloucester UK

**Keywords:** elevation models, photogrammetry, point cloud, reserve management, scrub

## Abstract

Scrub vegetation is a valuable habitat and resource for wildlife, but if unmanaged can encroach and dominate adjacent habitats, reducing biodiversity value. A primary task in the management of terrestrial nature reserves in the UK is monitoring and controlling scrub. The methods used to monitor and assess scrub cover are often basic, relying on qualitative assessment. Inaccurate assessments may fail to inform appropriate management of the habitats and lead to loss or degradation of important ecological features. Scrub can be monitored using UAV or satellite‐derived imagery, but it can be difficult to distinguish between other vegetation types without using high‐cost hyperspectral sensors. An alternative method using high‐resolution surface models from photogrammetric point clouds enables the isolation of vegetation types based on height. Scrub can be isolated from woodland, hedgerows, and tall ground vegetation. In this study, we calculate scrub cover using a photogrammetric point cloud modeling approach using UAVs.

We illustrate the method with two case studies from the UK. The scrub cover at Daneway Banks, a calcareous grassland site in Gloucestershire, was calculated at 21.8% of the site. The scrub cover at Flat Holm Island, a maritime grassland in the Severn Estuary, was calculated at 7%. This approach enabled the scrub layer to be readily measured and if required, modeled to provide a visual guide of what a projected management objective would look like. This approach provides a new tool in reserve management, enabling habitat management strategies to be informed, and progress toward objectives monitored.

## Graphical abstract

In this study, we demonstrate the use of UAV image analysis in modeling scrub vegetation cover as a useful tool in conservation management of nature reserves. Scrub, although valuable for wildlife, requires constant management and monitoring on nature reserves to maintain the favorable status of priority habitats such as unimproved grassland or ground nesting bird breeding habitats. In this study, we calculate scrub cover using a photogrammetric point cloud modelling approach using UAVs at two SSSIs in the UK.

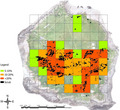

## 1 INTRODUCTION

Native scrub vegetation includes a range of forms from scattered bushes to closed canopy vegetation and represents valuable shelter and food resources for wildlife, particularly where it develops around highly valued priority habitats such as species‐rich grasslands, heath, and moorland (Mortimer et al., 2000). Scrub develops as an intermediate vegetation phase in the succession from open ground to close‐canopy woodland, usually up to around 5 m in height before it becomes woodland (JNCC, 2010). Despite its inherent advantages for wildlife, scrub can become undesirable when it encroaches and dominates priority habitats (Laborde & Thompson, 2015). Scrub management through grazing or manual removal is a challenging task for land managers in the UK, particularly in finding the right balance of maintaining scrub for biodiversity and ensuring protection of priority habitats or ecological features.

Priority habitats are often within sites covered by protective environmental legislation, which obliges the owner to maintain or restore the designated feature at “a favorable conservation status.” In the UK, the status of Sites of Special Scientific Interest (SSSIs), Special Protection Areas (SPAs), Special Areas of Conservation (SACs), or Ramsar sites are typically informed by Common Standards Monitoring (CSM), which provides a basic framework to ensure consistent monitoring of habitats, species, or geological features. For habitats, the CSM indicates the extent, vegetation structure, composition, and physical characteristics to reflect its condition. This often includes an indication of appropriate levels of scrub cover (typically referred to as a percentage, e.g., <5% cover for lowland calcareous grassland, JNCC, 2004a). For protected species groups, like ground nesting birds, an equivalent CSM assessment can include measuring the encroachment of scrub on available nesting habitat (JNCC, 2004b).

The assessment of scrub cover on such sites typically involves a “structured walk,” which involves following a route across the whole site, usually in a “W‐shape,” stopping at predetermined distances to make a local assessment. Such methods provide a rapid and useful assessment of the general situation, but do not generate an accurate representation of current scrub cover, or a robust means to compare with previous surveys.

The use of unmanned aerial vehicles (UAVs) can provide high‐resolution aerial imagery as a spectral basis to perform remote sensing (Zhang et al., 2016). It is possible to effectively monitor scrub vegetation using satellite‐derived imagery with remote sensing, particularly at broad scales where scrub is encroaching into open habitats like grasslands or savannahs (Laliberte et al., 2004; Mitchard et al., 2009). This approach can be more difficult at finer scales where either the resolution of satellite imagery is too broad to capture the vegetation patches (Marston et al., 2017) or the spectral qualities of scrub are too similar to the surrounding vegetation for it to be distinguishable (Redhead et al., 2012). Vegetation types are better distinguished with the use of specialist multispectral or hyperspectral sensors, which operate across the visible (400–700 nm), near‐infrared (700–1,300 nm), and short‐wave infrared (1,300–2,500 nm) portions of the electromagnetic spectrum (Fassnacht et al., 2016; JNCC, 2010). These sensors are well used in characterization, monitoring, and mapping of vegetation (Thenkabail & Lyon, 2016) and can be used to discriminate between different tree species (Jensen et al., 2012; Modzelewska et al., 2021).

It is also possible to make use of the spatial attributes of vegetation (such as its height) to distinguish scrub from ground vegetation and woodland. Vegetation elevation data can be derived from surface models generated using Light Detection and Ranging (LiDAR). This technology has been used as an alternative to field surveying, providing rapid accurate precise mapping across areas with challenging access (Bergen et al., 2009; Greaves et al., 2016). It has been used to detect and measure a range of ecological parameters such as forest canopy heights (Breidenbach et al., 2010; Burt et al., 2019; Genç et al., 2004) and the presence of scrub bushes (Streutker & Glenn, 2006). LiDAR can be acquired through satellite or UAV‐mounted LiDAR sensors, but both have substantial acquisition costs and require technical expertise that can limit the spatial and temporal extent on which they can be used (Wallace et al., 2012; Zarco‐Tejada et al., 2014).

It is possible to quickly and accurately identify vegetation heights using an establishing approach of structure‐from‐motion (SfM) photogrammetric point clouds derived from images from standard consumer‐grade UAVs (St‐Onge et al., 2015; Sperlich et al., 2014; Kattenborn et al., 2014; Birdal et al., 2017; Nevalainen et al., 2017; Cunliffe et al., 2016). This approach generates high‐resolution 3D models that are commonly used for geomatic applications such as measuring rock slope stability (Haneberg, 2008), the mapping and quantification of landforms (Westoby et al., 2012), and the biophysical structure of vegetation (Dunford et al., 2009; Dandois & Ellis, 2010). Most applications of point‐cloud models for mapping and measuring vegetation are for agroforestry systems for forest inventories and to quantify stock biomass (Iglhaut et al., 2019; Jayathunga et al., 2018), regeneration and recovery (Feduck et al., 2018; Goodbody et al., 2018), and tree growth (Mokroš et al., 2020), with few studies using this approach to characterize semi‐natural vegetation systems (Alonzo et al., 2020; Fraser et al., 2016; Rango et al., 2009).

In semi‐natural systems, single vegetation layers, such as grassland, scrub, or woodland, can be isolated from one another by categorizing the surface layer based on its typical height from the ground. In the case of scrub, this could be achieved by isolating vegetation with a surface height within the typical range for scrub (i.e., between 1 and 5 m). A similar approach of classifying semi‐natural vegetation types by their canopy height has been used with success in the calculation of biomass of shrub–grassland habitats in a dryland ecosystem (Cunliffe et al., 2016). In this study, we assess the use of a photogrammetric point‐cloud modeling approach to isolate and measure scrub cover in two SSSI nature reserves in the UK as a tool to measure existing scrub cover and inform management.

## 2 MATERIALS AND METHODS

### 2.1 Study sites

The study investigates two local nature reserves in the UK, Daneway Banks in Gloucestershire (UKGR SO939037) and Flat Holm Island in the Severn Estuary, Cardiff (UKGR ST221649).

Daneway Banks is a 16‐ha reserve supporting unimproved calcareous grassland and scattered blackthorn *Prunus spinosa* and hawthorn *Crataegus monogyna* scrub species across the site and boundaries. There is a small woodland consisting of beech *Fagus sylvatica*, yew *Taxus baccata*, and whitebeam *Sorbus aria* in the center of the site. The site is grazed by sheep and ponies to maintain a low grassland sward height. The scrub is a valued habitat on the site but is managed to minimize encroachment into the grassland through manual removal (Gloucestershire Wildlife Trust, 2013).

The terrestrial area of Flat Holm is 35 ha and supports important ecological features including cliff habitats, wild leek *Allium ampeloprasum*, and a large breeding colony of lesser black‐backed gull *Larus fuscus* (Natural Resources Wales, 2008). The northern half of the island is managed as a maritime grassland supporting very little scrub vegetation. The southern half of the island is unmanaged, resulting the extensive development of mature elder *Sambucus nigra* scrub. Within the current management plan, the scrub management objective states the target of maintaining mature scrub cover at a level less than 50% of the total land cover (Cardiff Harbour Authority, 2009).

### 2.2 Data collection

Two readily available consumer‐grade UAVs were used in this study. Daneway Banks was surveyed using a DJI T600 Inspire 1 quadcopter equipped with a 12 MP Zenmuse X3 RGB sensor. Flat Holm was surveyed using the DJI T900 Inspire 2 with 20 MP Zenmuse X4S RGB sensor. Both UAVs have comparable outputs, and choice of their use was driven by equipment availability and the greater stability of the Inspire 2 in more exposed conditions of the marine environment.

Transects were planned and conducted using Pix4D Capture (Pix4D China Technology Company) application running on a Sony Xperia android smartphone. The transects were programmed using the “double grid for 3D model” template, covering all areas of the site with front and side overlap setting of 80%. The cameras were angled at 70° (not nadir). The drone flying speed was set at “normal,” which was approximately 5 m/s. The shutter speed, ISO, and aperture for the both the Inspire 1 (Zenmuse X3) and Inspire 2 (Zenmuse X4S) were 1/2,000, 200, and f2.8, with focus set at infinity. At Daneway Banks, flights were undertaken on 3 July 2017 at an elevation above ground level of 50 m, giving a ground sample distance (GSD) of 2.49 cm/px. The image capture at Daneway Banks collected 1,127 images in five flights, which took 78 min of flight time. At Flat Holm, flights were conducted on 31 May 2019 at an elevation of 75 m, giving a GSD of 2.34 cm/px. The flight height was chosen on the basis of minimizing disturbance to the colony of lesser black‐backed gulls. The image capture at Flat Holm collected 1,417 images in 7 flights, which took 106 min of flight time. Images were saved on SD cards as tagged image file format (tiff) including the GPS position, camera orientation, and time.

### 2.3 Workflow

The data analysis workflow involved Generation of a photogrammetric point cloud and associated elevation models; Generation of an Above Ground Model (AGM); Defining the study boundary, Classification of height bands; and Measurement of the scrub layer.

### 2.4 Generation of a photogrammetric point cloud and associated elevation models

All UAV image files contain metadata of flight information (coordinates of UAV) and camera parameters (orientation, ISO, shutter speed, and aperture). All images were uploaded to Pix4D Mapper V 4.5.6, which automatically produces geo‐referenced orthomosaics and digital elevation models. Matching points are identified across all uploaded images, and their 3D coordinates are calculated using Structure from Motion algorithms. The points are interpolated to form a triangulated irregular network, which generates a dense point cloud. This point cloud enables all image pixels to be positioned in the same scale on an ortho‐rectified mosaic (or “orthomosaic”; Küng et al., 2012). In this study, we use the “3D Maps” standard template, which retains the full key points image scale in the initial processing. The point cloud densification was created at the original scale (1), at “optimal” point density, and a minimum of three matches for each point. As well as the orthomosaic, Pix4D generates a Digital Surface Model (DSM) and a Digital Terrain Model (DTM) as exportable raster tiff files.

#### 2.4.1 Generation of the Above Ground Model (AGM)

To isolate vegetation from the ground and eliminate the effect from topographical variation, the DTM was subtracted from the DSM (DSM‐DTM) using the raster calculator tool in ArcGIS Pro 2.5.2 (Esri Ltd.) to produce the Above Ground Model (AGM). The AGM comprises positive values of all pixels above the ground, representing ground vegetation, scrub, trees, and any other structures.

#### 2.4.2 Defining the study boundary

The site boundary and any other excluded features onsite (e.g., blocks of woodland) are manually defined as polygons and used to clip the AGM.

#### 2.4.3 Classification of height bands

The AGM is classified into three height bands including ground vegetation and flat surfaces (minimum pixel value to 1 m), mature scrub (1–5 m), and all vegetation and structures exceeding the scrub height (5 m to maximum pixel value). The values used to distinguish the scrub layers were verified on the ground at each site to ensure patches of low‐lying scrub and were actually scrub and not tall ruderal vegetation like bracken *Pteridium aquilinum*, nettle *Urtica dioca,* or willowherb *Epilobium* sp. The scrub layer at Daneway Banks was between 1.5 m and 5.75 m and between 1 m and 5 m at Flat Holm. The minimum size stand of vegetation to be classified as scrub was 0.5 m^2^ (0.25 cm × 0.25 cm). The accuracy of the classification was confirmed by physically visiting the stands on the ground with the scrub layer map.

#### 2.4.4 Measurement of the scrub layer

The scrub layer was isolated and converted into a polygon for area measurement. The area of each polygon is calculated in m^2^, which can be summarized as a total measurement for the whole site.

## 3 RESULTS

**FIGURE 1:**
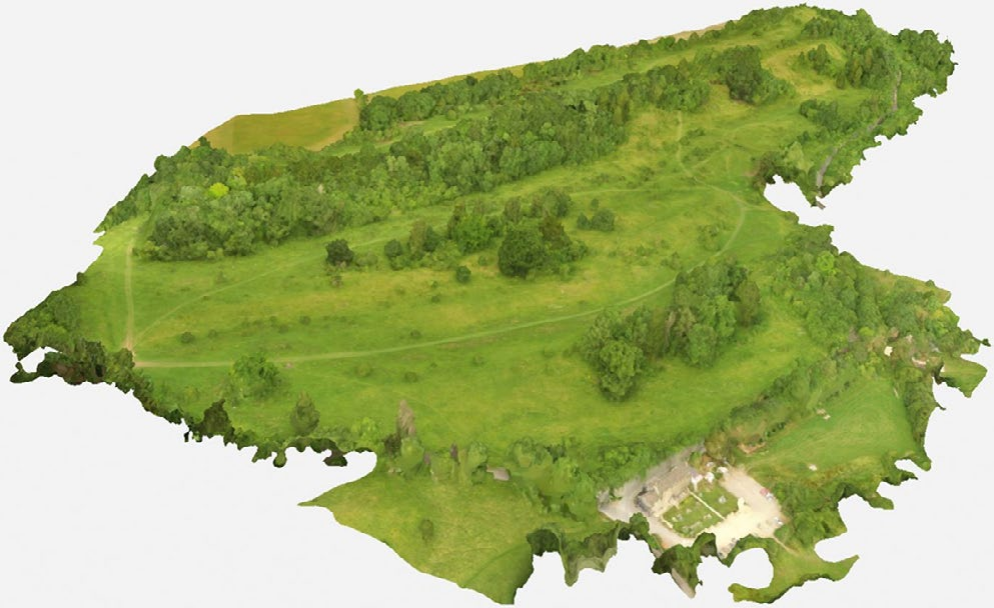
Photogrammetric point cloud‐derived 3D model of Daneway Banks

The Daneway Banks point cloud had a mean point density of 242.93 per m^3^ with a surface heights up to 16.7 m from ground level (Figure 1). DTM and DSM resolutions were 0.124 m^2^ and 0.025 m^2^. Vegetation classification was possible with minimal overlap, isolating scrub from tall grass and herbs, and trees. Ground verification confirmed that all scrub on the site was included in the model and no other vegetation types were classified as scrub.

**FIGURE 2:**
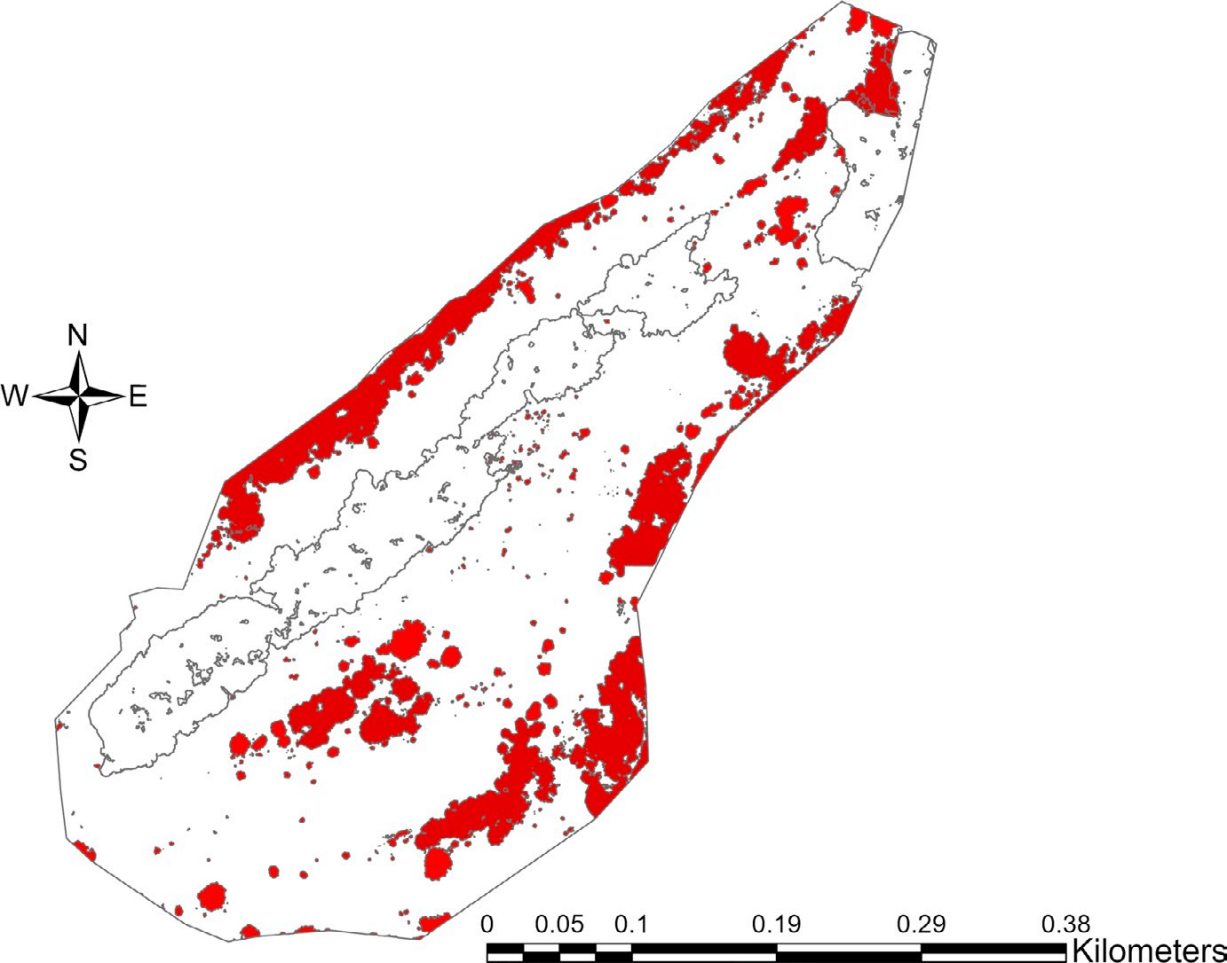
Scrub cover at Daneway Banks by classifying heights between 1 m and 5.75 m

Excluding the woodland habitat in the center of the site, the area of available calcareous grassland habitat at Daneway Banks is 130,920 m^2^. The calculated scrub layer comprised 391 separate stands ranging in area between 0.50 and 6,098 m^2^. The combined scrub cover on the site measured 28,352 m^2^ representing 21.65% cover of the calcareous grassland habitat (Figure 2).

**FIGURE 3:**
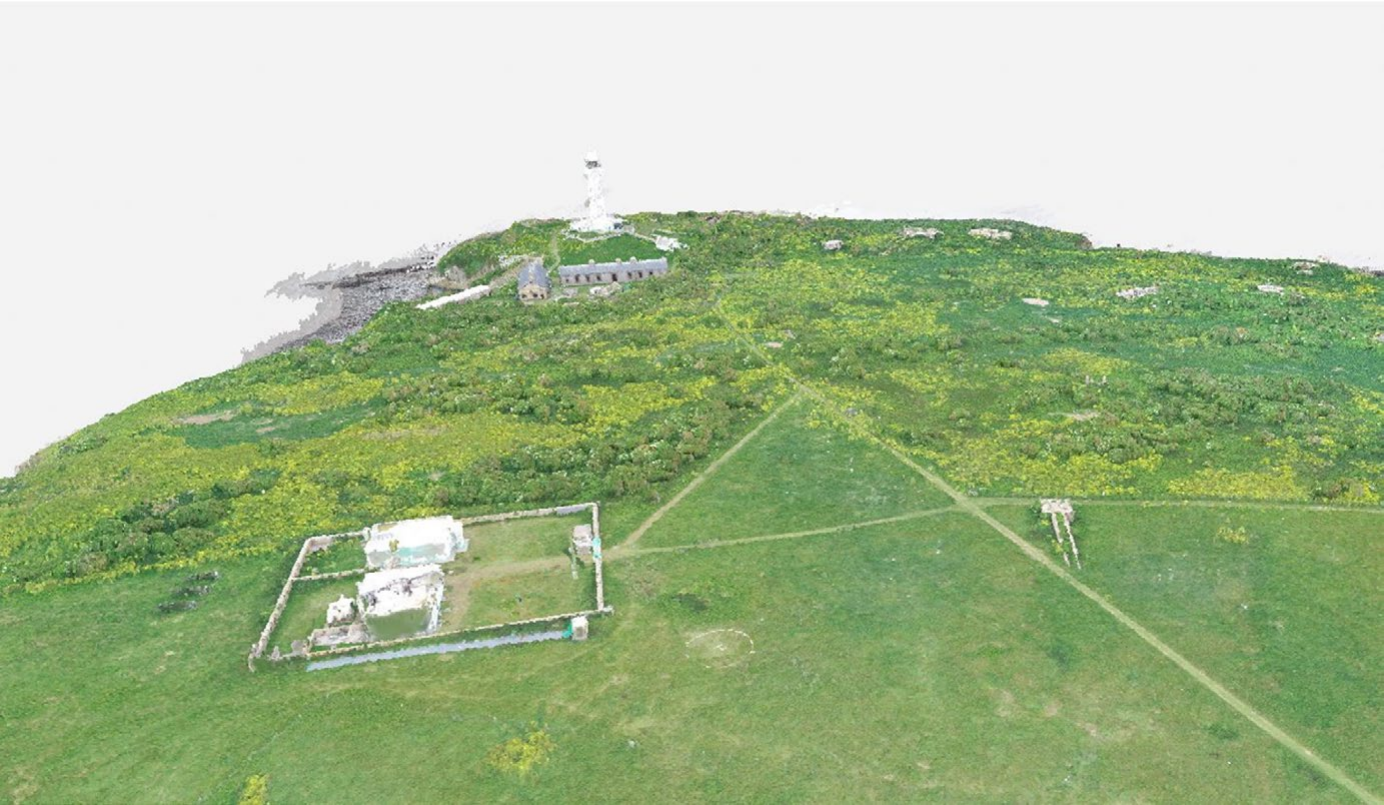
Photogrammetric point cloud‐derived 3D model of Flat Holm

**FIGURE 4:**
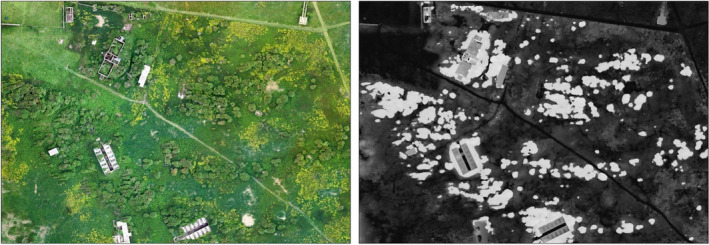
Isolation of the scrub layer and buildings on Flat Holm by classifying heights between 1.5 m and 5 m

The Flat Holm point cloud had a mean point density of 342.1 points per m^3^ with surface heights up to 29.48 m (the top of the lighthouse) and as low as −30 m (low tide foreshore; Figure 3). DTM and DSM resolutions were 0.117 m^2^ and 0.023 m^2^. Isolation of the scrub layer from the grass and tall herbs was possible using the height band between 1.5 and 5 m, although there was some overlap with low buildings, which required manual clipping (Figure 4).

**FIGURE 5:**
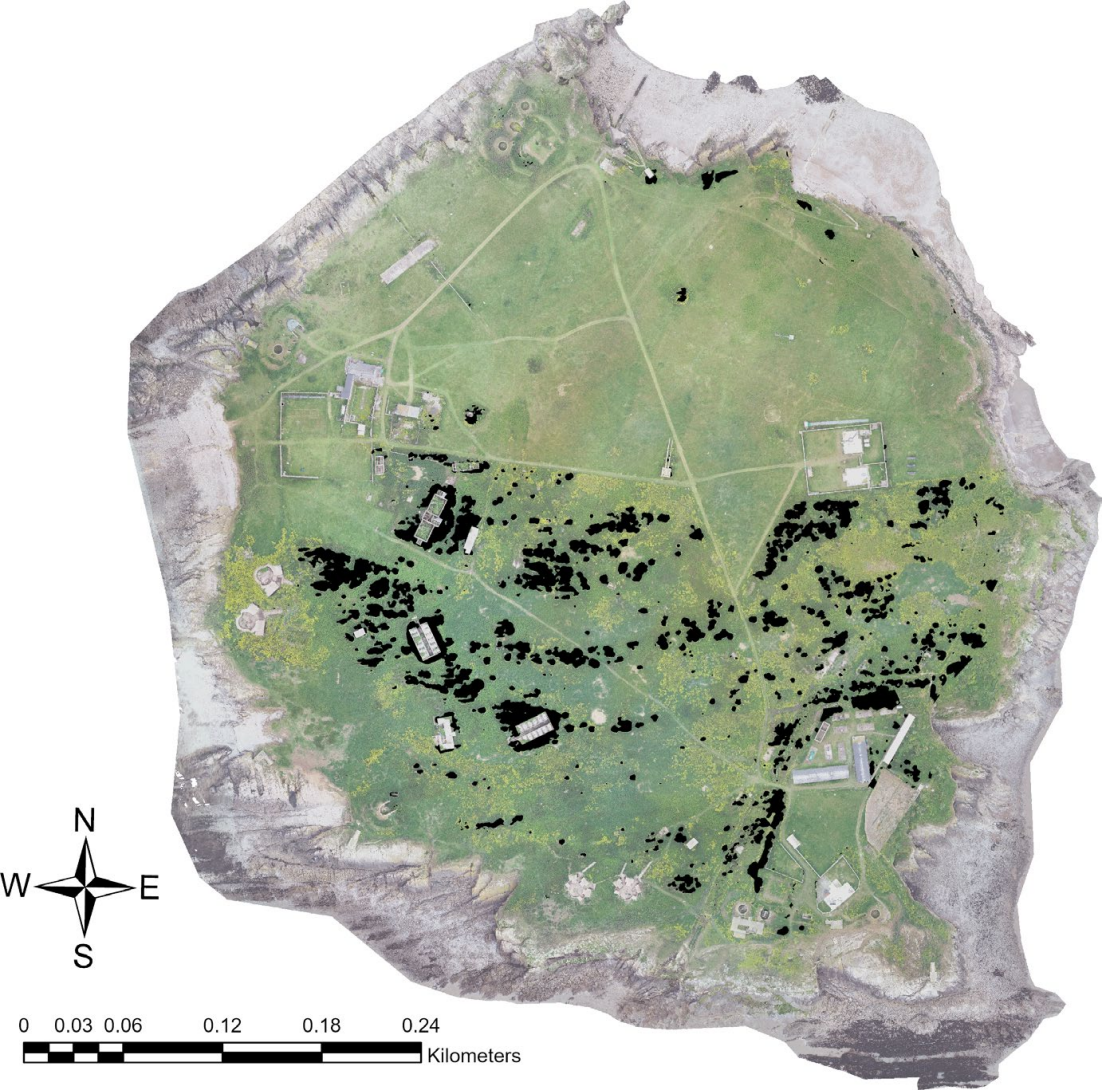
Total scrub cover on Flat Holm

Excluding cliff and beach habitat, buildings, and other functional spaces, the area of available terrestrial grassland habitat on Flat Holm is 172,723.93 m^2^. The process identified 501 stands of scrub ranging in size between 0.50 and 667.42 m^2^. The combined scrub cover was estimated as 12,067 m^2^ representing 7% of the available terrestrial grassland habitat (Figure 5). In the unmanaged southern half of the island, the mature scrub cover is 11,176 m^2^, representing 10.8% of the available terrestrial habitat.

## 4 DISCUSSION

In this study, a photogrammetric point‐cloud workflow was developed using consumer‐grade UAVs to classify and measure vegetation on the basis of height in two nature reserves. Using height as a basis for vegetation classification works well in this ecological surveying context as it correlates well with broad communities with limited height overlap. In both case studies, the scrub was scattered and distinct from other habitat types in height, so it was simple to provide precise results. The use of photogrammetry rather than LiDAR may be an effective alternative tool in applications where penetration of the subcanopy is not important. Comparisons with LiDAR have shown photogrammetry to provide equivalent accuracy in measuring structural attributes such as tree height and aboveground biomass (Filippelli et al., 2019). Similar approaches using photogrammetry have been used to map and measure geometric features in agricultural trees and forestry (Kattenborn et al., 2014; Ota et al., 2017; Torres‐Sánchez et al., 2018), and more studies increasingly used this technique to map and measure natural and semi‐natural vegetation on the basis of height (Alonzo et al., 2020; Fraser et al., 2016; Granholm et al., 2017; Cunliffe et al., 2016; Rango et al., 2009; Reese et al., 2015).

**FIGURE 6:**
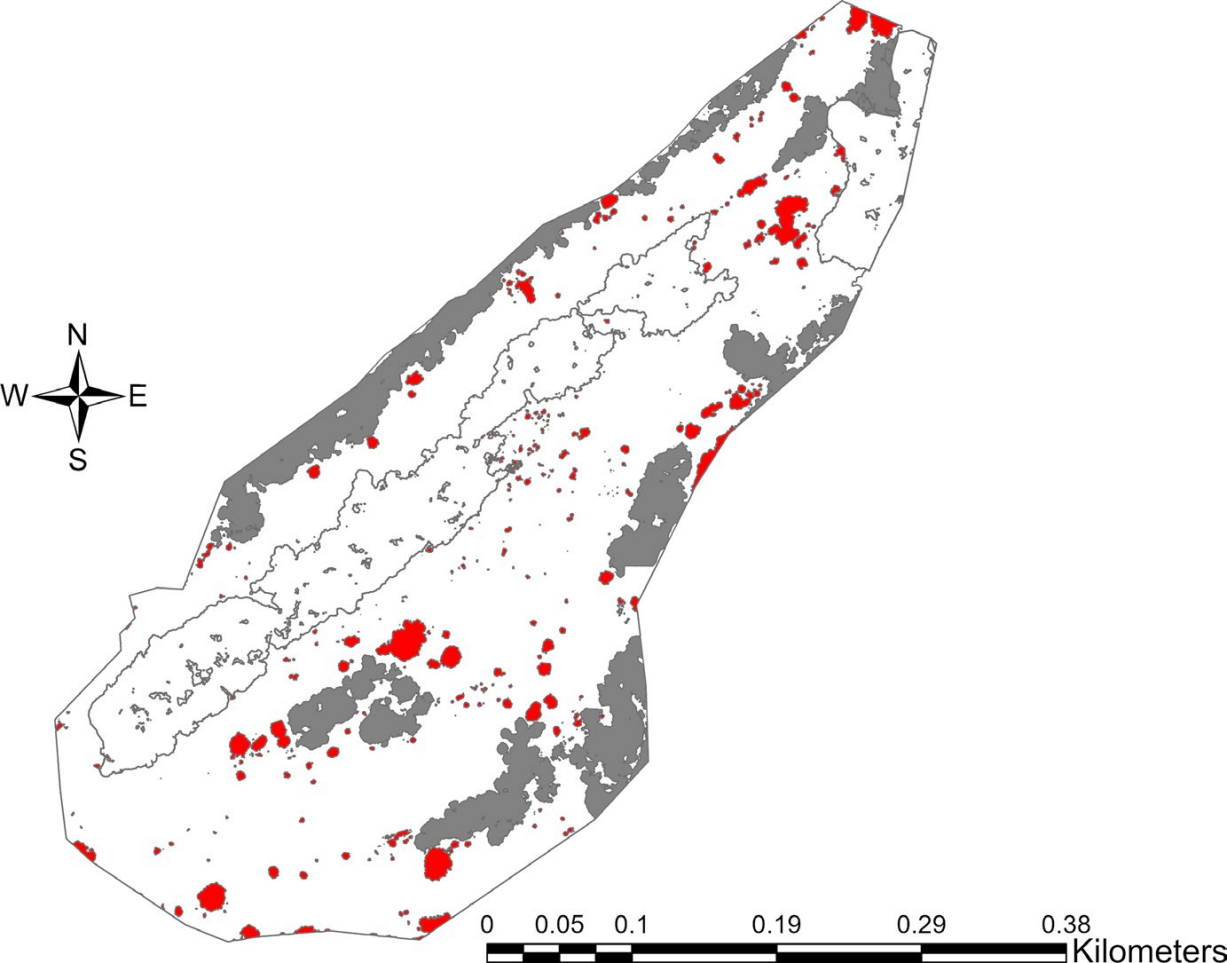
Scrub removal required at Daneway Banks to meet 5% management prescription (colored gray)

The value of precise scrub cover measurements in a conservation context is that these can be compared against management prescriptions, to visualize planned work, and monitor growth and reduction between years. For example, if the management of the grassland at Daneway Banks included a prescription that aligns with the CSM recommendation of maintaining no more than 5% scrub, we could use this approach to find that the current situation exceeds this by 16.77% or 21,500 m^2^ of excess scrub vegetation. Furthermore, we can use the existing scrub cover data to visualize what 5% scrub cover would look like on this site by identifying the stands to be removed (e.g., Figure 6). Progress toward management goals through scrub removal, as well as assessing scrub growth, could be achieved by undertaking survey and analysis annually.

At Flat Holm, the 11.4% scrub cover on the unmanaged south side of the island is far below the scrub cover prescribed in the management plan and was a lot lower than was estimated by the land managers, who assumed the level was closer to 50% and had planned scrub removal actions accordingly (pers. comm). The now known accurate scrub cover value can be used to inform and revise management targets in the context of other ecological features on the site such as the availability of breeding habitat for lesser black‐backed gulls (Ross‐Smith et al., 2013), safeguarding the position of endemic leek plants, or creating conditions for greater general biodiversity (Mortimer et al., 2000).

**FIGURE 7:**
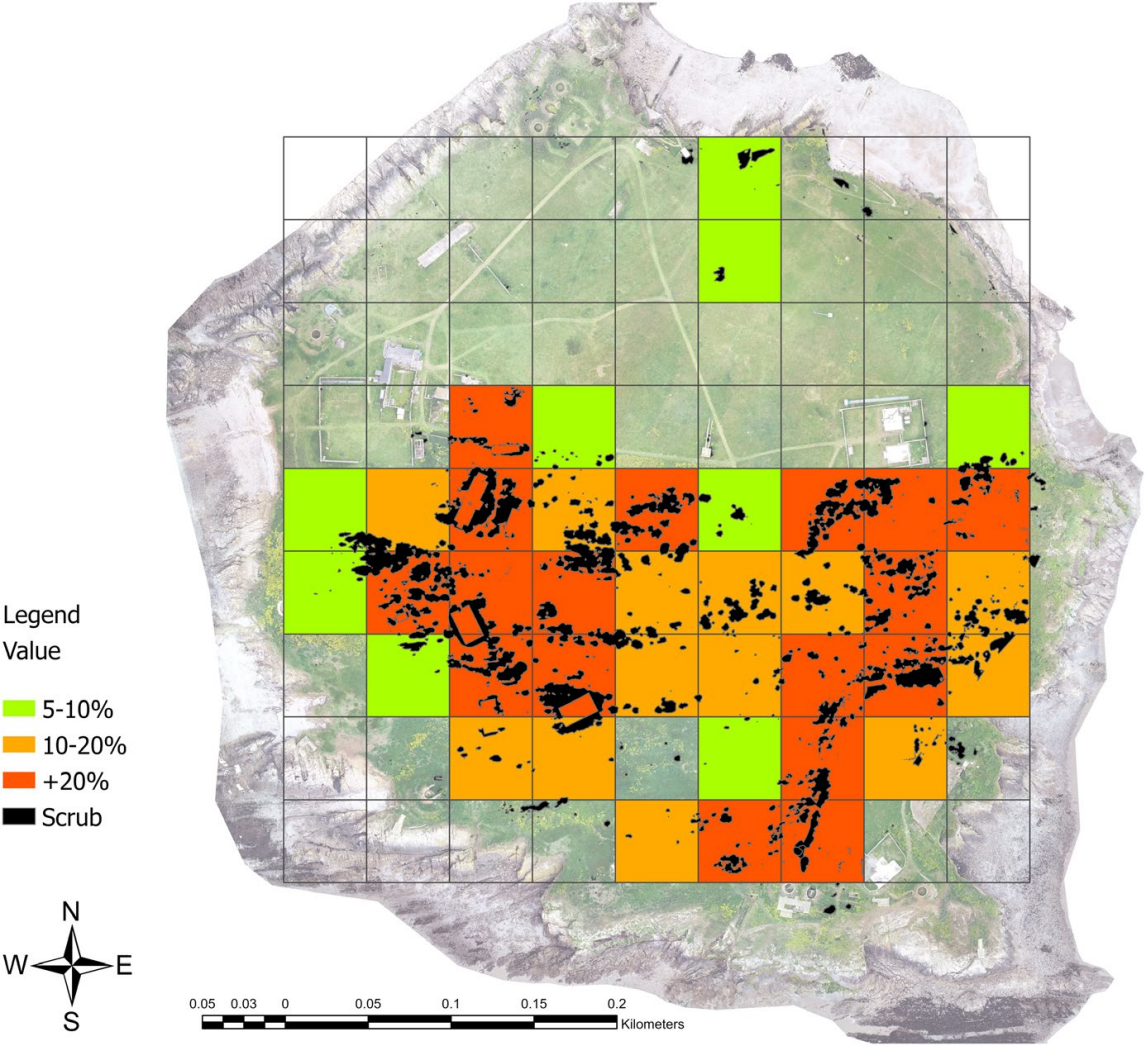
Assessment of scrub cover at Flat Holm at 50 m^2^ resolution

These outcomes might be achieved by maintaining a consistent level of scrub below a particular threshold or by maintaining a range of scrub conditions from dense patches to scattered scrub across the site. Both approaches could be informed by applying assessments across the site at higher resolutions (e.g., 50 m^2^ grids; Figure 7), which would highlight areas in need of management.

This study highlights that measurements of vegetation are possible at any scale and can be used to inform the prescriptions of CSMs and bespoke management plans. Accurate metrics are important to fulfill target and can inform the efficacy of management decisions and interventions (Rego et al., 2019). This study also highlights that the precise prescriptions in CSMs and management plans are impossible to accurately assess using ground‐based methods, and perhaps these were set at a time when traditional assessments should be taken as a rough guide.

In ecological habitat classification, we distinguish scrub from woodland by its height (i.e., by being less than 5 m; JNCC, 2010). When there is not substantial woodland habitat onsite, it is recommended that the height range be flexible to allow for taller scrub. This was found on Daneway Banks where scrub stands reached heights of 5.75 m, which would strictly be classified as woodland. In this context, classifying small patches of woodland are not appropriate. The isolation of scrub using a set height range risks excluding immature or low‐lying shrubs as well as those transitioning into woodland. Likewise, it risks including nonscrub vegetation like tall ruderal vegetation and bracken, or structures like buildings, fence‐lines, and vehicles. By further dividing the scrub layer into low lying (1–3 m) and taller (3–5), the taller scrub can be highlighted as more critical for management intervention. The use of simple height thresholds to isolate scrub could also be effective in other priority habitat types such as reedbeds and fens, heathlands, and upland river catchments. Although height is useful to determine vegetation classification, it requires validation using aerial imagery and some knowledge of local conditions to identify and exclude these issues (unless they feature as objects in infrastructure layers). In this way, photogrammetric point cloud, unlike LIDAR can integrate the spectral information with height information, which can inform and validate the classification process (Genç et al., 2004). Further work to combine this approach with multispectral sensors can further help classify vegetation to the species level.

The method used in this study is simple and mostly automated within analysis software and does not require extensive spatial analysis expertise and can be done by following a published protocol. The photogrammetric point‐cloud development and generation of the elevation models are well developed by PIX4D but are possible with other providers such as Dronedeploy, Esri ArcGIS Pro Orthomapping (Drone2map), IMAGINE Photogrammetry, and Agisoft Metashape. The subscription to the point‐cloud generation and GIS software is expensive (£700–1,000 per license) and maybe prohibitive for smaller organizations. The development of analysis workflows using opensource platforms such as QGIS, R, and Python is possible but require more substantial technical expertise. We undertook this study using the recommended default settings with regard to flight speed, overlap, and camera angle within the PIX4D capture app, to emulate the experience of a basic user.

Despite the investment in equipment, software and training, and the time involved in processing data, this approach demonstrates that precise measurements of scrub at the reserve level can be readily acquired and manipulated to generate management targets, monitor progress, and provide valuable insights into management objectives.

## CONFLICT OF INTEREST

No conflicts of interest.

## AUTHOR CONTRIBUTIONS

**Jim Vafidis:** Conceptualization (equal); data curation (lead); formal analysis (lead); project administration (equal); supervision (lead); validation (equal); visualization (equal); writing‐original draft (lead); writing‐review & editing (lead). **Isaac Lucksted:** Formal analysis (supporting); investigation (supporting); methodology (equal). **Moyrah Gall:** Formal analysis (supporting); investigation (equal). **Pete Maxfield:** Data curation (supporting); methodology (supporting). **Kathy Meakin:** Conceptualization (equal); validation (equal). **Mark Steer:** Conceptualization (equal); methodology (equal); writing‐review & editing (supporting).

## Data Availability

All data layers generated for both sites are available from Dryad https://doi.org/10.5061/dryad.0rxwdbs04.
